# Reduced intensity conditioning with 8 Gy total body irradiation in adult patients with acute lymphoblastic leukemia

**DOI:** 10.1038/s41409-025-02762-4

**Published:** 2025-12-06

**Authors:** Klaus Wethmar, Matthias Edinger, Kerstin Schäfer-Eckart, Matthias Stelljes, Thomas Schroeder, Kristina Sohlbach, Renate Arnold, Michael Stadler, Gesine Bug, Martin Bornhäuser, Gerald Wulf, Wolfgang Bethge, Edgar Jost, Daniel Teschner, Guido Kobbe, Monika Brüggemann, Lena Reiser, Dieter Hoelzer, Nicola Gökbuget, Stefan Schönland

**Affiliations:** 1 Department of Medicine A, Hematology, Oncology, Hemostaseology and Pneumology, University Hospital Münster, Münster, Germany; 2 Department of Internal Medicine III (Hematology and Oncology), University Hospital Regensburg, Regensburg, Germany and Leibniz-Institute for Immunotherapy, Regensburg, Germany; 3 Department of Internal Medicine 5, Klinikum Nürnberg, Paracelsus Medizinische Privatuniversität, Nürnberg, Germany; 4 Department of Hematology and Stem Cell Transplantation, University Hospital Essen, Essen, Germany; 5 Department of Internal Medicine, Hematology, Oncology and Immunology, University Hospital Giessen and Marburg, Marburg, Germany; 6 Hematology and Oncology, Charité-Universitätsmedizin Berlin, Berlin, Germany; 7 Hematology & Oncology, Medical Center University of Hannover, Hannover, Germany; 8 Goethe University, University Hospital, Department of Medicine II, Hematology/Oncology, Frankfurt, Germany; 9 Department of Internal Medicine I, Carl Gustav Carus University Hospital Dresden, TU Dresden, Dresden, Germany; 10 Department of Hematology and Oncology, Georg-August University Göttingen, Göttingen, Germany; 11 Department of Hematology, Oncology, Clinical Immunology and Rheumatology, University Hospital Tübingen, Tübingen, Germany; 12 Department of Hematology, Oncology, Hemostaseology and Stem Cell Transplantation, University Hospital RWTH Aachen, Aachen, Germany; 13 Department of Internal Medicine II, University Hospital Würzburg, Würzburg, Germany; 14 Department of Hematology, Oncology and Clinical Immunology, University Hospital Düsseldorf, Düsseldorf, Germany; 15 Department of Internal Medicine II, University Hospital Schleswig-Holstein, Kiel, Germany; 16 Department of Medicine V, University of Heidelberg, Heidelberg, Germany

**Keywords:** Acute lymphocytic leukaemia, Clinical trials

## Abstract

Total body irradiation (TBI) plus chemotherapy is commonly applied prior to allogeneic hematopoietic stem cell transplantation (SCT) for patients with acute lymphoblastic leukemia (ALL). Here, we retrospectively analyzed registry data from the German Multicenter Study Group for Adult ALL (GMALL) and report the outcomes for 111 adult ALL patients who received 8 Gy TBI-based SCT conditioning in first complete remission between 2002 and 2018 after initial treatment with pediatric-based approaches. Patients had a median age of 52 years (range 18–65) at initial diagnosis, and the majority of patients (93%) had a good performance status (ECOG 0/1). 97 patients (87%) showed high-risk features according to GMALL criteria, of whom 58 (60%) were Philadelphia chromosome/*BCR::ABL1*-positive. With a median follow-up of 3.1 years after SCT, the survival rates at one, three, and five years were 72%, 64%, and 57% for disease-free survival, and 76%, 67%, and 61% for overall survival, respectively. The rates of non-relapse mortality at one, three, and five years were 22%, 26%, and 30%, while the cumulative incidences of relapse were 7%, 10%, and 14%, respectively. In summary, 8 Gy TBI conditioning in ALL patients was feasible and resulted in outcomes similar to those previously reported for 12 Gy conditioning regimens.

## Introduction

Allogeneic hematopoietic stem cell transplantation (SCT) is part of the current standard of care for patients with high-risk acute lymphoblastic leukemia. In young adults (<45 years) without significant comorbidities 12 Gray total body irradiation (TBI) together with cyclophosphamide (Cy) or etoposide (VP16) is recommended within the treatment protocols of the German Multicenter Study Group for Acute Lymphoblastic Leukemia (GMALL) prior to SCT.

Due to increased toxicity of the 12 Gy conditioning approach in older and/or comorbid patients, reduced-intensity TBI-based conditioning regimens (RIC), mostly 8 Gy TBI plus fludarabine (FLU), or TBI-free chemotherapy-based conditioning using FLU and busulfan (BU) or FLU and melphalan (MEL), have been applied with only minor differences in patients´ outcomes [1, 2]. However, in a recent retrospective analysis based on registry data of the European Society for Blood and Marrow Transplantation (EBMT), reduced intensity TBI-based regimens compared favorably to chemotherapy-based conditioning [3]. Another retrospective EBMT study compared the outcome of patients receiving 12 Gy versus 8 Gy TBI-based conditioning, both in combination with FLU chemotherapy, and did not identify significant differences with respect to overall survival (OS), disease-free survival (DFS), non-relapse mortality (NRM), and cumulative incidence of relapse (CIR) [4]. Intermediate-dose TBI/fludarabine conditioning can also be applied in patients with other hematopoietic malignancies, such as peripheral T-cell lymphoma, resulting in a favorable toxicity/efficacy profile [5]. Considering these data and additional reports evaluating various other TBI- and non-TBI-based conditioning regimes [6, 7], the impact of TBI dosing for the outcome of ALL patients after SCT remains a matter of debate and no generally accepted conditioning regimen has been established.

Here, we retrospectively analyzed GMALL registry and clinical trial data of patients who received 8 Gy reduced-intensity TBI-based conditioning prior to SCT to evaluate patient characteristics, transplant-related features and outcome parameters in a homogeneously treated ALL cohort. Due to the observed increased NRM in patients aged 45 or older [2], TBI 8 Gy with FLU was the recommended conditioning for this age group in GMALL protocols. All patients received first-line therapy according to pediatric-based approaches. The transplant indication was based on uniform GMALL risk criteria and the proposed time for SCT was after two phases of induction and first consolidation.

## Patients and methods

### Patients

This retrospective analysis included 111 adult patients (>18 years of age) with ALL, who received conditioning chemotherapy together with 8 Gy TBI prior to SCT between February 2002 and December 2018. Inclusion criteria were defined as follows: 1) diagnosis of acute lymphoblastic leukemia (ALL); 2) Treatment according to GMALL first-line protocols; 3) age ≥18 years; 4) SCT in first complete remission (CR1).

GMALL registry data was analyzed to extract patient-, donor-, treatment- and transplant-characteristics as well as follow-up and outcome parameters. Measurable residual disease (MRD) was determined by immunoglobulin/T-cell receptor rearrangement-specific quantitative polymerase chain reaction [IG/TR qPCR] for at least one leukemia-specific IG/TR gene rearrangement or another leukemia-specific genetic aberration with a sensitivity of at least 10^−4^. Molecular complete remission (molCR) was assumed if the MRD result was negative or below 10^−4^.

### Treatment

Defining the current study cohort, pre-transplant conditioning for all patients was based on 8 Gy TBI. Patients received pediatric-inspired induction and consolidation regimens within GMALL trials (06/99, NCT00199056 or 07/03, NCT00198991) or subsequent similar treatment recommendations documented in the prospective GMALL registry (NCT02872987). 8 Gy TBI plus FLU was recommended for patients >45 years of age within the GMALL protocols. However, younger patients could also receive 8 Gy TBI at the discretion of the treating physician, primarily due to significant comorbidities or pre-treatment toxicities.

Induction regimens for Philadelphia chromosome/BCR::ABL1-negative ALL consisted of vincristine, daunorubicine, (peg-)asparaginase, dexamethasone, 6-mercaptopurine, cytarabine, and cyclophosphamide. Consolidation I regimens contained high-dose methotrexate, dexamethasone, vindesine, etoposide and high-dose cytarabine. CD20 positive B-ALL patients received four doses of rituximab during induction and consolidation I. All patients received intrathecal prophylaxis (methotrexate with or without cytarabine and dexamethasone) and 24 Gy CNS radiotherapy. Patients with Philadelphia chromosome/BCR::ABL1-positive ALL received induction therapy containing vincristine, dexamethasone, (peg-)asparaginase and consolidation I as described above, both together with continuous oral imatinib.

SCT in CR1 was generally recommended in GMALL protocols for high-risk patients up to the age of 55, and could be performed for older patients at the discretion of the treating physician.

### Outcome measures

Data are presented as percentages for discrete variables and medians for continuous variables. Distribution of discrete variables was compared with the chi-square test to detect significant differences. OS was measured as time between the date of SCT and the date of death from any cause with right-censoring of surviving patients at the last follow-up date. DFS was measured as time between the date of SCT and the date of hematologic relapse or date of death from any cause, whatever occurred first. Surviving patients in continuous remission were right-censored at the last follow-up date. OS and DFS were calculated by Kaplan–Meier product-limit-estimator and heterogeneity of the time-to-event distribution functions was calculated by log-rank test (*p* values <0.05 were considered as significant). The cumulative incidences of relapse were calculated from the date of SCT to the date of hematologic relapse with right-censoring for patients in continuous remission at the last follow-up date. NRM was calculated as cumulative incidence from the date of SCT to the date of death from any cause with right-censoring of surviving patients at the last follow-up date. For the calculation of the cumulative incidence of relapse, death from any reason was considered as a competing event, whereas hematologic relapse was considered as competing event for the calculation of non-relapse mortality. The time-to-event distribution functions of cause-specific cumulative incidences across strata were compared by Gray’s test. All statistical analysis and presentation were performed using Statistical Analysis Software (release 9.4) procedures and macros (SAS/STAT User’s Guide 14.3; Cary, NC).

## Results

### Patient characteristics

We retrospectively analyzed the outcomes of 111 adult patients with acute lymphoblastic leukemia, who received SCT conditioning based on 8 Gy total body irradiation between February 2002 and December 2018 following induction/first consolidation according to GMALL protocols. The study included 66 male and 45 female patients with a median age of 52 years at initial diagnosis (range 18–65 years), with the majority of patients (77%) being 46 years or older (Table 1). The ECOG performance status was 0 or 1 for 93% of patients and 87% of patients had documented high risk features according to the GMALL criteria, which include >30,000 leukocytes/µl in B-precursor ALL, no complete hematologic remission (CR or CRi) after the first induction cycle (day 22), pro-B-ALL or translocation t(4;11), early or mature T-ALL, and t(9;22)/Philadelphia chromosome/*BCR::ABL1* (Ph+ ALL). Persistence or recurrence of MRD was later integrated as an indication for SCT. In the subgroup of documented high-risk ALLs (*n* = 97) 60% of patients had a Ph+ ALL, 15% had a non-thymic-T-ALL, 13% a pro-B-ALL, 6% had a late CR beyond first induction, and 5% had initial leukocyte blood counts of more than 30,000 leukocytes/µl. The majority of all patients (83%) were transplanted after receiving two cycles of induction therapy and one cycle of consolidation therapy as scheduled according to the GMALL protocols. 7% were transplanted after induction II and 4% after consolidation II. Overall, the median time to SCT was 166 days after diagnosis. For the Ph+ ALL subgroup (*n* = 58), information on post-SCT TKI administration was available for 28 cases. Of these, 13 patients received post-SCT TKIs for inconsistent medical reasons, which prevented further analysis.

**Table 1:** Patient and transplantation characteristics.

Patients	MSD (*n* = 31)		MUD (*n* = 80)		*p*^b^	Total (*n* = 111)	
	*n*	%	*n*	%		*n*	%
Median age, years (range)	51 (22–61)		53 (18–65)			52 (18–65)	
Age groups (*n* = 111)							
18–45 yrs	7	12%	19	24%	>0.05	26	23%
>45 yrs	24	77%	61	76%		85	77%
Sex (*n* = 111)							
male	18	58%	48	60%	>0.05	66	59%
female	13	42%	32	40%		45	41%
Subtype (*n* = 109)							
c/pre B	8	26%	12	15%	>0.05	20	18%
pro B	3	10%	10	13%		13	12%
early T	6	19%	5	6%		11	10%
mature T	2	6%	2	3%		4	4%
Ph+	12	39%	46	59%		58	53%
other	0	0%	3	4%		3	3%
High risk features^a^ (*n* = 97)							
WBC c/pre B	3	11%	2	3%	>0.05	5	5%
late CR c/pre B	2	7%	4	6%		6	6%
pro B	3	11%	10	14%		13	13%
early T	6	21%	5	7%		11	11%
mature T	2	7%	2	3%		4	4%
Ph+	12	43%	46	67%		58	60%
Median time to SCT, days (range)	164 (121–350)		166 (112–702)			166 (112–702)	
Time of SCT (*n* = 111)							
after induction II	2	6%	6	8%	>0.05	8	7%
after consolidation I	26	84%	66	83%		92	83%
after consolidation II	0	0%	4	5%		4	4%
later	3	10%	4	5%		7	6%
MRD status at SCT (*n* = 33)							
molecluar CR	7	64%	10	45%	>0.05	17	52%
molecular failure	4	36%	12	55%		16	48%
ECOG status at SCT (*n* = 61)							
0	6	40%	19	41%	>0.05	25	41%
1	7	47%	25	54%		32	52%
2	2	13%	2	4%		4	7%
Matching (*n* = 92)							
identical	26	100%	50	76%	**0.005**	76	83%
one mismatch	0	0%	16	24%		16	17%
Conditioning (*n* = 111)							
TBI/fludarabin	27	87%	65	81%	>0.05	92	83%
other^c^	4	13%	15	19%		19	17%
GvHD prophylaxis (*n* = 89)							
CsA, MTX	10	45%	35	52%	>0.05	45	51%
CsA, MTX, other	8	36%	14	20%		22	25%
other	4	18%	18	26%		22	25%
Sex of donor (D) – recipient (R) (*n* = 69)							
male D - female R	3	14%	10	21%	**0.024**	13	19%
female D - male R	9	41%	5	11%		14	20%
female D - female R	5	23%	9	19%		14	20%
male D - male R	5	23%	23	49%		28	41%
Age of donor (D) – recipient (R) (*n* = 57)							
D ≤ 35/R ≤ 35	2	10%	1	3%	**0.0002**	3	5%
D ≤ 35/R > 35	2	10%	23	62%		25	44%
D > 35/R ≤ 35	0	0%	0	0%		0	0%
D > 35/R > 35	16	80%	13	35%		29	51%
CMV status of donor (D) – recipient (R) (*n* = 86)							
negative D – negative R	2	10%	24	37%	**0.0001**	26	30%
negative D – positive R	0	0%	16	25%		16	19%
positive D – positive R	14	67%	20	31%		34	40%
positive D – negative R	5	24%	5	8%		10	12%

*MSD* matched sibling donor, *MUD* matched unrelated donor, *MM* mismatch, *yrs* years, *WBC* white blood cell, *CR* complete remission, *SCT* stem cell transplantation, *ECOG* Eastern Cooperative Oncology Group, *MRD* minimal residual disease, *pos* positive, *TBI* total body irradiation, *GvHD* graft-versus-host disease, *CsA* Cyclosporine A, *MTX* methotrexate, *D* donor, *R* recipient, *CMV* cytomegalovirus.

^a^No data on KMT2A rearrangements.

^b^*p* Chi² (if necessary Fisher’s Exact Test): distribution MSD vs MUD, significant values are highlighted in bold.

^c^Other: 1 TBI; 1 TBI/CP; 7 TBI/CP/ATG; 4 TBI/CP/fludarabin; 5 TBI/CP/fludarabin/ATG; 1 TBI/VP16/ATG.

### Allogeneic hematopoietic stem cell transplantation

Of the 111 ALL patients receiving 8 Gy TBI, 92 (83%) were treated with concomitant fludarabine chemotherapy conditioning. The remaining 19 patients received no (*n* = 1) or various other types of accompanying chemotherapy, including eight and nine patients who received TBI together with cyclophosphamide alone or a combination of cyclophosphamide and fludarabine, respectively, and one patient receiving TBI and etoposide (Table 1). Allogeneic grafts were collected from 31 matched sibling donors (MSD) and 80 matched unrelated donors (MUD). Exact donor information was available for 92 cases, including 26 HLA-identical MSD, 50 HLA-identical MUD and 16 MUD with one mismatching HLA allele. Approximately 75% of patients received prophylactic ciclosporin A (CSA) and methotrexate (MTX) to prevent Graft versus Host Disease (GvHD), including 25% of patients receiving additional immunosuppressive agents. The remaining quarter of patients received other GvHD prophylaxis without CSA/MTX. Approximately half of the donors with documented age (*n* = 57) were younger than 35 years, while 95% of recipients were beyond 35 years of age. Within the MUD subgroup, the proportion of donors <35 years of age was higher compared to the MSD subgroup (*p* = 0.0002).

### Response and outcome

**Fig. 1:**
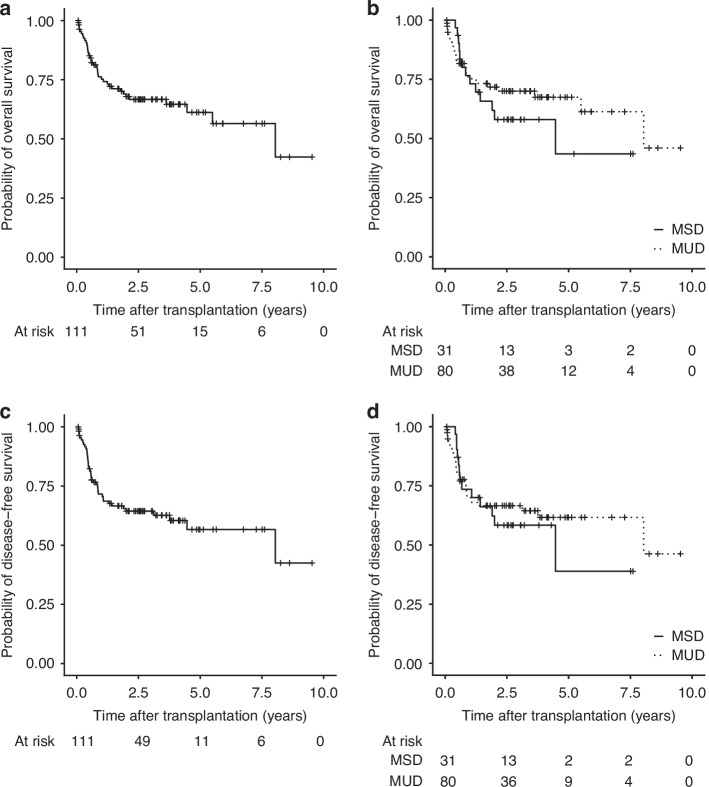
Overall survival (OS) and disease-free survival (DFS). Kaplan–Meier estimates of **a** OS in the entire cohort, **b** OS in the MSD (solid line) and MUD (dashed line) subgroups, **c** DFS in the entire cohort, **d** DFS in the MSD (solid line) and MUD (dashed line) subgroups.

**Fig. 2:**
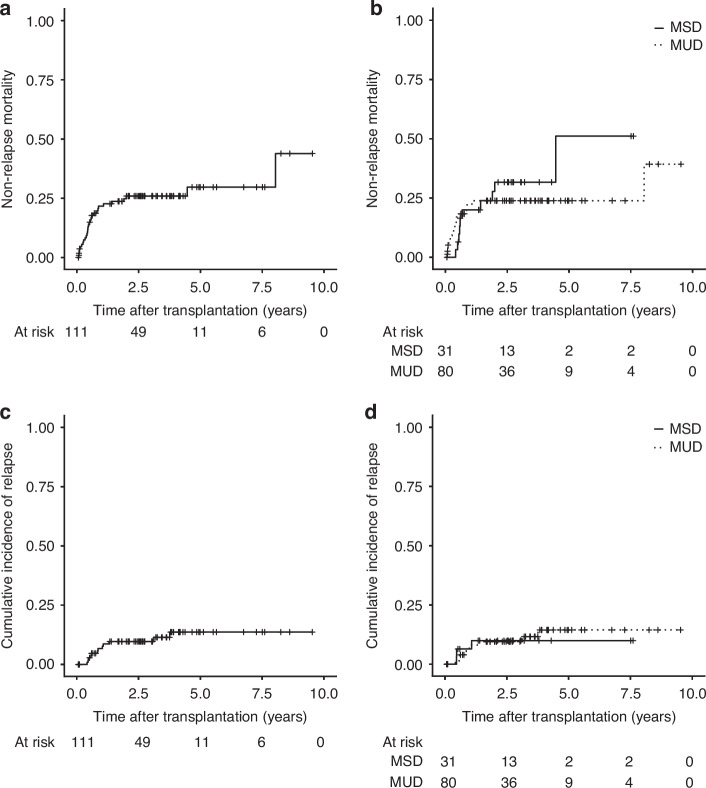
Non-relapse mortality (NRM) and relapse risk (RR). **a** NRM in the entire cohort, **b** NRM in MSD (solid line) and MUD (dashed line) subgroups, **c** RR in the entire cohort, **d** RR in MSD (solid line) and MUD (dashed line) subgroups.

The median follow-up time since SCT was 3.1 years (range 0.06 to 9.53 years). The OS of the entire study cohort was 76% at one year, 67% at three years and 61% at 5 years after SCT (Fig. 1 and Supplementary Table S1). DFS rates were 72% at one year, 64% at three years and 57% at 5 years after SCT. NRM for the entire cohort was 22%, 26%, and 30% at one, three, and five years of follow-up and the risk of relapse (RR) was 7%, 10%, and 14%, respectively (Fig. 2 and Supplementary Table S1). No differences in outcome measures were observed when we compared MSD and MUD cohorts (Figs. 1 and 2, and Supplementary Table S1). Of note, patients who received SCT after the year 2010 had improved OS and DFS compared to those who were transplanted between 2002 and 2010 (Supplementary Table S1).

A higher risk of early mortality until day 100 after SCT (*p* = 0.033) was observed in patients receiving MUD grafts (8/80 cases) versus MSD grafts (0/31 cases). Overall, 8/29 (28%) of non-relapse-related deaths occurred within the first 100 days after transplant, while 52% of NRM was observed within the remaining first and 14% in the second year after SCT. Infections with (21%) or without (48%) concurrent GvHD were the most common cause of NRM reported (Supplementary Table S2). At data cut-off 12 patients had relapsed, 7 (58%) within the first year after SCT, 3 (25%) during the second year, and 2 (17%) beyond two years after SCT.

Looking at the univariate impact of various subgroup characteristics on OS at three years after SCT (Table 2), we detected better OS rates (82% vs 56%, *p* = 0.046) for patients receiving fully matching MUD grafts as compared to those receiving mismatching MUD grafts (max. 1 mismatch). Within the limited subgroup of MSD recipients (*n* = 31), OS at three years was better among patients experiencing no or grade I acute GvHD versus grad II-IV acute GvHD (*p* = 0.0017) and for those receiving 8 Gy TBI plus FLU versus other conditioning regimens (*p* = 0.017). However, both of these effects were not observed for MUD recipients and the overall cohort, respectively. 16 patients with documented absence of chronic GvHD and 22 patients with documented limited chronic GvHD showed excellent long-time survival (94% and 95% at three years, respectively) as compared with those experiencing extensive GvHD (71%, *p* = 0.004). All other subgroups, including age, sex, ALL subtype, high-risk features, ECOG status, MRD prior to SCT, and time of SCT had no significant effect on three-year OS rates in the overall study population (Supplementary Table S3).

**Table 2:** Subgroup analysis of 3-year overall survival (OS).

Patients	MSD (*n* = 31)			MUD (*n* = 80)			Total (*n* = 111)		
	*n*	OS	*p*^a^	*n*	OS	*p*^a^	*n*	OS	*p*^a^
Age									
18–45 yrs	7	43%	>0.05	19	77%	>0.05	26	68%	>0.05
>45 yrs	24	64%		61	68%		85	67%	
Matching									
Identical^b^	26	62%	–	50	82%	**0.046**	76	75%	>0.05
One mismatch	0	–		16	56%		16	56%	
Conditioning									
TBI/Flu	27	63%	**0.017**	65	71%	>0.05	92	68%	>0.05
Other^c^	4	25%		15	67%		19	58%	
Acute GvHD^d^									
Grade 0-I	16	67%	**0.0017**	45	79%	>0.05	61	75%	>0.05
Grade II-IV	5	0%		9	78%		14	50%	
Chronic GvHD^e^									
No	1	100%	>0.05	15	93%	**0.041**	16	94%	**0.004**
Limited	6	80%		16	100%		22	95%	
Extensive	4	50%		3	100%		7	71%	

*MSD* matched sibling donor, *MUD* matched unrelated donor, *yrs* years, *OS* overall survival.

^a^*p* Log Rank: impact of subgroup, significant values are highlighted in bold.

^b^HLA identical (6/6, 8/8, 10/10).

^c^Other: 1 TBI; 1 TBI/CP; 7 TBI/CP/ATG; 4 TBI/CP/Fludarabin; 5 TBI/CP/Fludarabin/ATG; 1 TBI/VP16/ATG.

^d^Landmark analysis at day +100 post SCT

^e^Landmark analysis at day +180 post SCT.

Patients >45 years of age showed increased 3-year NRM of 30% versus 12% for younger patients (*p* = 0.038), while inversely, the 3-year RR was lower (5% vs. 24%, *p* = 0.005) (Table 3). With the limitation of small patient numbers, NRM was higher among patients encountering acute GvHD grade II-IV (*n* = 14) versus those without or grade I acute GvHD (*n* = 61), reaching 50% and 13% (*p* = 0.005), respectively. The subgroup of patients with documented extensive chronic GvHD showed a numerical trend towards higher NRM and RR versus patients without or limited chronic GvHD (*p* > 0.05), while all other subgroups, as reported for the OS analysis, did not reveal significant differences in NRM or RR (Supplementary Tables S4, S5).

**Table 3:** Subgroup analysis of 3-year non-relapse mortality (NRM) and relapse risk (RR).

Subgroup	MSD (*n* = 31)			MUD (*n* = 80)			Total (*n* = 111)		
	*n*	NRM	*p*^a^	*n*	NRM	*p*^a^	*n*	NRM	*p*^a^
Age									
18–45 yrs	7	14%	>0.05	19	11%	>0.05	26	12%	**0.038**
>45 yrs	24	36%		61	28%		85	30%	
Matching									
identical	26	34%	–	50	15%	>0.05	76	22%	>0.05
one mismatch	0	–		16	31%		16	31%	
Conditioning									
TBI/Fludarabin	27	33%	>0.05	65	22%	>0.05	92	25%	>0.05
other^b^	4	25%		15	33%		19	32%	
Acute GvHD^c^									
grade 0–I	16	13%	**0.0006**	45	14%	>0.05	61	13%	**0.005**
grade II–IV	5	100%		9	22%		14	50%	
Chronic GvHD^d^									
no	1	0%	>0.05	15	0%	>0.05	16	0%	>0.05
limited	6	20%		16	0%		22	5%	
extensive	3	33%		3	0%		6	17%	

Subgroup	*n*	RR	*p*^a^	*n*	RR	*p*^a^	*n*	RR	*p*^a^
Age									
18–45 yrs	7	43%	**0.001**	19	17%	>0.05	26	24%	**0.005**
>45 yrs	24	0%		61	7%		85	5%	
Matching									
identical	26	4%		50	9%	>0.05	76	7%	>0.05
one mismatch	0	–		16	13%		16	13%	
Conditioning									
TBI/Fludarabin	27	4%	**0.003**	65	10%	>0.05	92	8%	>0.05
other^b^	4	50%		15	7%		19	16%	
Acute GvHD^c^									
grade 0–I	16	19%	>0.05	45	14%	>0.05	61	15%	>0.05
grade II–IV	5	0%		9	0%		14	0%	
Chronic GvHD^d^									
no	1	0%	>0.05	15	7%	>0.05	16	6%	>0.05
limited	6	0%		16	6%		22	5%	
extensive	3	0%		3	33%		6	17%	

*NRM* non-relapse mortality, *RR* relapse risk, *MSD* matched sibling donor, *MUD* matched unrelated donor, *yrs* years, *TBI* total body irradiation, *GvHD* Graft-versus-host disease.

^a^*p* Gray Test, significant values are highlighted in bold.

^b^Other: 1 TBI; 1 TBI/CP; 7 TBI/CP/ATG; 4 TBI/CP/Fludarabin; 5 TBI/CP/Fludarabin/ATG; 1 TBI/VP16/ATG.

^c^Landmark analysis at day +100 post SCT.

^d^Landmark analysis at day +180 post SCT.

## Discussion

Reducing treatment intensity in an older or vulnerable patient population appears to be inevitable, but comes at the cost of higher risk for relapse of the disease [8]. In our current non-comparative retrospective analysis, we report the outcome of adult patients with acute lymphoblastic leukemia who received RIC based on 8 Gy TBI prior SCT in CR1 after standardized induction and early consolidation therapy according to the GMALL protocols. The observed rates of 67% OS and 64% DFS after three years are comparable to the previously reported EBMT data for FLU/TBI-based conditioning with respect to both, 8 Gy and 12 Gy TBI-based regimens [4]. Of note, the NRM rate of 26% after two years of follow-up appeared to be somewhat higher in our GMALL cohort as compared to approximately 18% NRM being reported for the 8 Gy TBI EBMT cohort, while relapse-rates were lower (10% GMALL vs 21% EBMT).

Similar outcomes were also reported by another EBMT analysis among older ALL patients (>45 years) who received 8 Gy TBI-Flu conditioning [3]. Here, the two-year rates of 69% OS, 58% leukemia-free survival, 17% NRM, and 25% CIR after 8 Gy TBI-Flu compared favorably with two FLU/BU-based chemo-only-conditioning regimens. In another large retrospective analysis by the EBMT, TBI-based conditionings were also associated with improved outcomes in patients with both, MRD-negative and MRD-positive status at SCT [9]. For children and adolescents (4–21 years) with high-risk or relapsed ALL who were transplanted in CR1-3, conditioning with fractionated 12 Gy TBI and etoposide yielded significantly better results than chemotherapy-based conditioning with fludarabine, thiotepa, and either busulfan or treosulfan [10]. Similar observations were reported from a recent meta-analysis, where TBI-based and chemotherapy-based conditioning regimens were evaluated in >5500 pediatric and adolescent patients (0–24 years of age), with TBI-based regimens being associated with better OS (relative risk [RR] 1.21), better event-free survival (RR 1.34), and reduced risk of relapse (RR 0.69) [11]. In a recent EBMT retrospective cohort of 427 adult ALL patients, better LFS and lower NRM was also observed for TBI-based myeloablative conditioning (MAC) in the setting of haploidentical SCT with post-transplant cyclophosphamide, while there was no difference in relapse rates and OS between TBI- and CT-based MAC [12]. Somewhat conflicting data comes from a recent Chinese trial that compared the outcome of standard-risk adult ALL patients who were transplanted in CR1 and received 2 × 4.5 Gy TBI plus cyclophosphamide (TBI/Cy) versus bususlfan/cyclophosphamide (Bu/Cy)-based conditioning [13]. The data showed non-inferiority of the Bu/Cy-based conditioning in terms of efficiency and safety as compared to TBI/Cy. However, this study has limited impact for countries, where SCT is reserved for high-risk and Ph-chromosome/*BCR::ABL1*-positive ALL only.

Chemo-based conditioning may be an option for patients who are ineligible for TBI, as reported by a phase 2 trial (NCT00682305) evaluating the use of treosulfan, etoposide and cyclophosphamide prior to SCT, but the estimated 2-year disease-free and overall survival rates of 36% and 48%, respectively, and a CIR of 51% after 2 years clearly demonstrated the limited efficacy of this approach [14]. Further comparisons of TBI- versus chemo-based approaches come from a recent meta-analysis summarizing the outcome of more than 4700 patients >/=16 years of age of 7 additional studies [15], supporting the current standard of TBI-based conditioning regimens in patients with ALL by demonstrating better OS (hazard ratio 0.76) and PFS (hazard ratio 0.74) in the TBI cohorts.

The current study has several limitations. The study cohort consists of a limited number of patients that was recruited over a long period of time and was analyzed retrospectively. The registry data was incomplete, even for relevant subgroup-defining information, such as the ECOG performance status, donor matching, MRD level, comorbidities, and long-term toxicities. The current GMALL registry now includes more detailed instructions for documenting these variables in electronic case report forms, aiming to collect more robust data for future analyses. For the cohort reported in this study, patient treatment apart from 8 Gy TBI conditioning was subject to major changes in conventional ALL therapy and significant advances in supportive care. This is also reflected by our observation that the overall outcomes for patients in terms of OS and DFS have improved for the cohort treated after 2010. Refined pre-SCT regimens integrating rituximab, pediatric-inspired protocols, intensification of (pegylated)-Asparaginase, molecular MRD-guided treatment, as well as antibody-based salvage therapy with blinatumomab and inotuzumab ozogamicin, post-SCT supportive care (e.g. CMV prophylaxis), and the use post-SCT TKI for patients with Ph+ ALL may have collectively contributed to this positive development in ALL treatment [16, 17]. However, as an example, in our study actual data on post-SCT TKI use was available for only 28 of the 58 Ph+ patients, preventing conclusive further analysis. Other univariate subgroup analyses revealed some effects for HLA-matching, type of conditioning, as well as acute and chronic GvHD. However, patient numbers were again too small to deduct firm conclusions or to perform reliable multivariate calculations. In our cohort, there was no difference of OS among younger patients vs. those above 45 years of age. However, we did observe lower overall NRM and higher overall RR for younger patients. This is likely due in part to the significant comorbidities experienced by younger patients who received 8 Gy instead of 12 Gy of TBI. These comorbidities may have prompted the decision to administer reduced-intensity TBI, as well as reduced-intensity pre-transplant chemotherapy induction and consolidation. The higher NRM and lower RR among patients >45 years of age may also have been influenced by the high proportion (65%) of younger donors (<35 years) in the MUD subgroup, as was previously suggested for a CIBMTR cohort [18]. However, larger cohorts of patients are needed to accurately analyze the impact of individual patient-related variables and changes in ALL treatment in a multivariate fashion.

In conclusion, our and previous analyses indicate that RIC based on 8 Gy TBI and FLU is feasible in high-risk ALL patients and is part of the current GMALL recommendation for patients older than 45 years. Further trials including pediatric patients will investigate whether a reduction of TBI dose will be feasible in terms of NRM and relapse risk in younger patients as well.

## Supplementary information

Supplementary material

## Data Availability

The data that support the findings of this study are available through the GMALL Study Center upon reasonable request.
