# Supplementary material for: Reduced intensity conditioning with 8 Gy total body irradiation in adult patients with acute lymphoblastic leukemia
Source: Bone Marrow Transplant. 2025 Dec 6;61(3):303–11. doi: 10.1038/s41409-025-02762-4 (PMC12965881; doi:10.1038/s41409-025-02762-4)
Supplement: Supplementary file 1 — Supplementary material [file 41409_2025_2762_MOESM1_ESM.pdf]

# - Supplementary material -

Reduced intensity conditioning with 8 Gy total body irradiation in Adult Patients with Acute Lymphoblastic Leukemia

Klaus Wethmar[1], Matthias Edinger[2], Kerstin Schäfer-Eckart[3], Matthias Stelljes[1], Thomas Schroeder[4], Kristina Sohlbach[5], Renate Arnold[6], Michael Stadler[7], Gesine Bug[8], Martin Bornhäuser[9], Gerald Wulf[10], Wolfgang Bethge[11], Edgar Jost[12], Daniel Teschner[13], Guido Kobbe[14], Monika Brüggemann[15], Lena Reiser[8], Dieter Hoelzer[8], Nicola Gökbuget[8,*], Stefan Schönland[16,*].

Affiliations:

[1]Department of Medicine A, Hematology, Oncology, Hemostaseology and Pneumology, University Hospital Münster, Münster, Germany; [2]Department of Internal Medicine III (Hematology and Oncology), University Hospital Regensburg, Regensburg, Germany and Leibniz-Institute for Immunotherapy, Regensburg, Germany; [3]Department of Internal Medicine 5, Klinikum Nürnberg, Paracelsus Medizinische Privatuniversität, Nürnberg, Germany; [4]Department of Hematology and Stem Cell Transplantation, University Hospital Essen, Essen, Germany; [5]Department of Internal Medicine, Hematology, Oncology and Immunology, University Hospital Giessen and Marburg, Marburg, Germany; [6]Hematology and Oncology, Charité-Universitätsmedizin Berlin, Berlin, Germany; [7]Hematology & Oncology, Medical Center University of Hannover, Hannover, Germany; [8]Goethe University, University Hospital, Department of Medicine II, Hematology/Oncology, Frankfurt, Germany; [9]Department of Internal Medicine I, Carl Gustav Carus University Hospital Dresden, TU Dresden, Germany; [10]Department of Hematology and Oncology, Georg-August University Göttingen, Göttingen, Germany; [11]Department of Hematology, Oncology, Clinical Immunology and Rheumatology, University Hospital Tübingen, Tübingen, Germany; [12]Department of Hematology, Oncology, Hemostaseology and Stem Cell Transplantation, University Hospital RWTH Aachen, Aachen, Germany; [13]Department of Internal Medicine II, University Hospital Würzburg, Würzburg, Germany; [14]Department of Hematology, Oncology and Clinical Immunology, University Hospital Düsseldorf, Düsseldorf, Germany; [15]Department of Internal Medicine II, University Hospital Schleswig-Holstein, Kiel, Germany; [16]Department of Medicine V, University of Heidelberg, Heidelberg, Germany.

*N.G. and S.S. contributed equally to this work.

Short title: 8 Gy TBI conditioning for ALL

Table S1, Overall outcome at one, three, and five years.

	**MSD (n=31)**	**MUD (n=80)**	**p***	**2002-2010 (n=31)**	**2011-2018 (n=80)**	**p***	**Total (n=111)**
**Overall survival**							
1 year	77%	76%	.36	61%	82%	**.02**	76%
3 years	58%	70%		55%	72%		67%
5 years	44%	67%		47%	72%		61%
**Disease free survival**							
1 year	74%	71%	.60	55%	79%	**.03**	72%
3 years	58%	67%		52%	69%		64%
5 years	39%	62%		44%	66%		57%
**Non-relapse mortality**							
1 year	20%	22%	.47	35%	16%	>.05	22%
3 years	32%	24%		35%	22%		26%
5 years	51%	24%		40%	22%		30%
**Relapse risk**							
1 year	6%	7%	.89	10%	5%	>.05	7%
3 years	10%	10%		13%	8%		10%
5 years	10%	14%		17%	11%		14%

*p Logrank-test: distribution MSD vs MUD.

Table S2, Causes of NRM

**Patients with NRM**	**MSD (n=10)**		**MUD (n=19)**			**Total (n=29)**	
	**n**	**%**	**n**	**%**	**p***	**n**	**%**
**Causes of NRM**							
Infection	3	30%	11	58%	>.05	14	48%
Infection, GvHD-related	4	40%	2	11%		6	21%
Other	2	20%	4	21%		6	21%
Unknown	1	10%	2	11%		3	10%

Abbreviations: NRM, non-relapse mortality; MSD, matched sibling donor; MUD, matched unrelated donor; GvHD, graft-versus-host disease. *p Log Rank: impact of subgroup.

Table S3, Subgroup analysis of 3-year overall survival (OS) - continued

	MSD			MUD			entire cohort		
**Patients, n**	**31**			**80**			**111**		
	**patients**	**OS**	**p***	**patients**	**OS**	**p***	**patients**	**OS**	**p***
**Sex**									
Male	18	53 %	>.05	48	66 %	>.05	66	62 %	>.05
Female	13	66 %		32	76 %		45	73 %	
**Subtype**									
common/Pre-B	8	40 %	>.05	12	82 %	>.05	20	66 %	>.05
Pro B	3	67 %		11	60 %		13	62 %	
Early T	6	44 %		5	80 %		11	60 %	
Mature T	2	50 %		2	100 %		4	75 %	
Ph+	12	74 %		46	69 %		58	70 %	
Other	0	-		3	67 %		3	67 %	
**High risk feature**									
WBC c/pre B	3	67 %	>.05	2	100 %	>.05	5	80 %	>.05
Late CR c/pre B	2	50 %		4	75 %		6	67 %	
Pro B	3	67 %		10	60 %		13	62 %	
Early T	6	44 %		5	80 %		11	60 %	
Mature T	2	50 %		2	100 %		4	75 %	
Ph+	12	74 %		46	69 %		58	70 %	
**ECOG status**									
0	6	83 %	>.05	19	76 %	>.05	25	78 %	>.05
1	7	38 %		25	52 %		32	50 %	
2	2	100 %		2	100 %		4	100 %	
**MRD at week 16**									
Mol CR	7	69 %	>.05	10	100 %	>.05	17	87 %	>.05
Mol Failure	4	50 %		12	83 %		16	74 %	
**Time of SCT**									
After induction	2	50 %	>.05	6	50 %	>.05	8	50 %	>.05
After Cons I	26	66 %		66	70 %		92	69 %	
After Cons II	0	-		4	67 %		4	67 %	
Later	3	0 %		4	100 %		7	44 %	

Abbreviations: MSD, matched sibling donor; MUD, matched unrelated donor; yr/yrs, year/years; WBC, white blood cell; CR, complete remission; SCT, stem cell transplantation; ECOG, Eastern Cooperative Oncology Group; MRD, minimal residual disease; pos, positive; Mol, molecular; Cons, consolidation; TBI, total body irradiation; GvHD, Graft-versus-host disease. *p Log Rank: impact of subgroup.

Table S4, Subgroup analysis of 3-year non-relapse mortality (NRM) - continued

Patients, n	MSD 31			MUD 80			entire cohort 111		
	patients	NRM	p*	patients	NRM	p*	patients	NRM	p*
**Sex**									
Male	18	41%	>.05	48	27%	>.05	66	31%	>.05
Female	13	17%		32	20%		45	19%	
**Subtype**									
common/Pre-B	8	46%	>.05	12	9%	>.05	20	23%	>.05
Pro B	3	33%		10	30%		13	31%	
Early T	6	33%		5	0%		11	18%	
Mature T	2	50%		2	0%		4	25%	
Ph+	12	18%		46	28%		58	26%	
Other	0			3	33%		3	33%	
**High risk feature**									
WBC c/pre B	3	33%	>.05	2	0%	>.05	5	20%	>.05
Late CR c/pre B	2	50%		4	0%		6	17%	
Pro B	3	33%		10	30%		13	31%	
Early T	6	33%		5	0%		11	18%	
Mature T	2	50%		2	0%		4	25%	
Ph+	12	18%		46	28%		58	26%	
**ECOG status**									
0	6	17%	>.05	19	17%	>.05	25	17%	>.05
1	7	48%		25	38%		32	39%	
2	2	0%		2	0%		4	0%	
**MRD at week 16**									
Mol CR	7	31%	>.05	10	0%	>.05	17	13%	>.05
Mol Failure	4	25%		12	17%		16	19%	
**Time of SCT**									
After induction	2	50%	>.05	6	50%	>.05	8	50%	>.05
After Cons I	26	25%		66	22%		92	23%	
After Cons II	0	-		4	33%		4	33%	
Later	3	67%		4	0%		7	39%	

Abbreviations: NRM, non-relapse mortality; RR, relapse risk; MSD, matched sibling donor; MUD, matched unrelated donor; yr/yrs, year/years; WBC, white blood cell; CR, complete remission; SCT, stem cell transplantation; ECOG, Eastern Cooperative Oncology Group; MRD, minimal residual disease; Mol, molecular; pos, positive; Cons, consolidation; TBI, total body irradiation; GvHD, Graft-versus-host disease. NRM described as cumulative incidences at 3 years. *p Gray Test. **Other: 1 TBI; 1 TBI/CP; 7 TBI/CP/ATG; 4 TBI/CP/Fludarabin; 5 TBI/CP/Fludarabin/ATG; 1 TBI/VP16/ATG.

Table S5, Subgroup analysis of 3-year relapse risk (RR) - continued

	MSD (n=31)			MUD (n=80)			Total (n=111)		
**Patients**	**n**	**RR**	***p****	**n**	**RR**	**p***	**n**	**RR**	**p***
**Sex**									
Male	18	6%	>.05	48	14%	>.05	66	12%	>.05
Female	13	15%		32	3%		45	7%	
**Subtype**									
c/pre B	8	13%	>.05	12	9%	>.05	20	11%	>.05
pro B	3	0%		10	10%		13	8%	
early T	6	22%		5	20%		11	20%	
mature T	2	0%		2	0%		4	0%	
Ph+	12	8%		46	7%		58	7%	
other	0	-		3	67%		3	67%	
**High risk feature**									
WBC c/pre B	3	0%	>.05	2	0%	>.05	5	0%	>.05
late CR c/pre B	2	0%		4	25%		6	17%	
pro B	3	0%		10	10%		13	8%	
early T	6	22%		5	20%		11	20%	
mature T	2	0%		2	0%		4	0%	
Ph+	12	8%		46	7%		58	7%	
**ECOG**									
0	6	0%	>.05	19	13%	>.05	25	9%	>.05
1	7	14%		25	13%		32	13%	
2	2	0%		2	0%		4	0%	
**MRD**									
molecular CR	7	0%	>.05	10	11%	>.05	17	7%	>.05
molecular failure	4	25%		12	0%		16	7%	
**Time of SCT**									
after induction II	2	0%	>.05	6	17%	>.05	8	13%	>.05
after consolidation I	26	8%		66	8%		92	8%	
after consolidation II	0	-		4	33%		4	33%	
later	3	33%		4	0%		7	17%	

Abbreviations: RR, relapse risk; MSD, matched sibling donor; MUD, matched unrelated donor; yr/yrs, year/years; WBC, white blood cell count; CR, complete remission; SCT, stem cell transplantation; ECOG, Eastern Cooperative Oncology Group; MRD, minimal residual disease; pos, positive; TBI, total body irradiation; GvHD, Graft-versus-host disease. *p Gray Test. **Other: 1 TBI; 1 TBI/CP; 7 TBI/CP/ATG; 4 TBI/CP/Fludarabin; 5 TBI/CP/Fludarabin/ATG; 1 TBI/VP16/ATG.
